# Greenhouse gas emissions from ditches in oil palm plantations on tropical peatlands in Malaysia

**DOI:** 10.1038/s41598-025-21094-3

**Published:** 2025-10-23

**Authors:** Kuno Kasak, Iryna Dronova, Kaido Soosaar, Lulie Melling, Wong Guan Xhuan, Faustina Sangok, Reti Ranniku, Jorge A. Villa, Sheel Bansal, Michael Peacock, Ülo Mander

**Affiliations:** 1 Department of Geography, University of Tartu, Tartu, Estonia; 2 Department of Environmental Science, Policy, and Management, University of California, Berkeley, USA; 3 Sarawak Tropical Peat Research Institute, Sarawak, Malaysia; 4 University of Louisiana at Lafayette, Lafayette, LA USA; 5 U.S. Geological Survey, Northern Prairie Wildlife Research Center, Jamestown, ND USA; 6 Department of Aquatic Sciences and Assessment, Swedish University of Agricultural Sciences, Uppsala, Sweden; 7 Department of Geography and Planning, University of Liverpool, Liverpool, UK

**Keywords:** Tropical peatlands, Oil palm plantations, Carbon balance, Nitrous oxide, Methane ebullition, Upscaling, Biogeochemistry, Environmental sciences

## Abstract

Tropical peatlands, which store 20% of global peat carbon, are increasingly threatened by conversion to alternative land-uses such as oil palm plantations, pulp wood plantations, crop growth or other economic activities. This transformation involves peatland drainage, which lowers water tables, exposes peat to oxygen, and alters greenhouse gas (GHG) emissions: increasing carbon dioxide (CO_2_) and nitrous oxide (N_2_O) fluxes while reducing methane (CH_4_) emissions from soils. However, drainage ditches created in the process may become significant sources of CH_4_ due to anoxic conditions. This study quantified GHG fluxes from drainage ditches in Sarawak, Malaysia, through spatial sampling conducted during the daytime in the transitional period between the drier and wetter seasons using portable trace gas analyzers. Median fluxes were 0.19 g CH_4_ m^−2^ d^−1^, 17.1 g CO_2_ m^−2^ d^−1^, and − 0.12 mg N_2_O m^−2^ d^−1^. Physical water parameters such as pH, oxygen concentration, temperature, and oxidation–reduction potential were found to be significant drivers of GHG fluxes. The median emissions from ditches in one hectare of land were 5.84 kg CO_2_ ha^−1^ d^−1^, 2.78 kg CH_4_ as CO_2_ eq ha^−1^ d^−1^, and − 0.001 kg N_2_O as CO_2_ eq ha^−1^ d^−1^. These findings underscore the role of drainage ditches as CH_4_ sources in tropical peatland agriculture, highlighting the need for further research into GHG management in these modified landscapes.

**Supplementary Information:**

The online version contains supplementary material available at 10.1038/s41598-025-21094-3.

## Introduction

Tropical peatlands are among the world’s most threatened ecosystems while storing approximately 20% of global peatland carbon^1–4^. However, their carbon storage capacity is increasingly threatened by extensive ditching, drainage, and agricultural conversion, particularly to oil palm plantations. Further, despite their potentially high greenhouse gas (GHG) emissions^5^, fluxes from drainage ditches in tropical peatlands are poorly documented, hindered by limited access to plantations at various conversion stages. Filling the knowledge gap on ditch GHG emissions is critical to accurately assess and constrain the global warming potential of past, current, and future tropical peatland conversions.

In Southeast Asia, which holds approximately 40% of the world’s tropical peatlands, widespread land conversion has occurred over the last three decades^6,7^. Tropical peatlands are primarily drained for agriculture and plantation activities, lowering the water table and exposing previously accumulated peat organic material to oxygen. The resulting aerobic decomposition releases carbon dioxide (CO_2_) and contributes to land subsidence^8^. Moreover, drained peatlands can become notable sources of powerful GHGs like nitrous oxide (N_2_O)^9–11^, and may continue to emit methane (CH_4_)^12^. Both Cooper et al.^13^ and Deshmukh et al.^9^ noted that, when considering all three GHGs (CO_2_, CH_4_, and N_2_O), the conversion of intact tropical peat swamp forests to oil palm plantations can at least double total soil GHG emissions. Therefore, lowering the groundwater level can substantially increase the CO_2_ and N_2_O emissions to the atmosphere.

After land conversion through drainage, CH_4_ emissions are generally expected to decrease as the soils become exposed to oxygen, which reduces methanogenesis in the topsoil layer^14^. For example, Wong et al.^12^ showed that oil palm plantations had significantly lower CH_4_ emissions than undrained peat swamp forests. However, while CH_4_ emissions from drained soils may decrease, the emissions from newly created drainage ditches can become important alternate pathways for CH_4_ production and release due to their anoxic conditions, warm temperatures, and high availability of carbon^15^. Yet, only a few studies to date have documented GHG fluxes from ditches on peat soil in the tropics^5,16–18^. These studies have shown large variations in CO_2_ and CH_4_ fluxes, and therefore calculating accurate emissions factors for ditches in oil palm plantations remains challenging; the current Intergovernmental Panel on Climate Change (IPCC) CH_4_ emission factor for ditches in drained tropical peatlands (2259 kg CH_4_ ha^−1^ yr^−1^) relies on a single study^5,19^. In addition, none of these studies have partitioned the total CH_4_ flux into diffusive and ebullitive flux. Partitioning CH_4_ emission into diffusive and ebullitive is essential for accurately quantifying total CH_4_ emissions^20^. Ebullition is highly episodic, releasing small or large bursts of CH_4_ in short events, whereas diffusion is continuous^21^. This episodic nature means that relying on one type of emission pathway might overlook major emissions during ebullition events, skewing overall CH_4_ flux estimates. Moreover, models aiming to estimate regional or global CH_4_ emissions need to capture the variability between diffusive and ebullitive pathways. Diffusive emissions can be more predictable based on temperature and other stable conditions, but ebullitive emissions can add a layer of unpredictability that needs to be accounted for^22^. Furthermore, information on N_2_O emissions from drainage ditches in tropical peatlands is sparse, with studies like Jauhiainen and Silvennoinen^5^ being among the few to document N_2_O fluxes. This underscores a critical need for studies that address CH_4_ emission pathways and revisit and quantify N_2_O emissions under current environmental conditions and management practices.

**Fig. 1:**
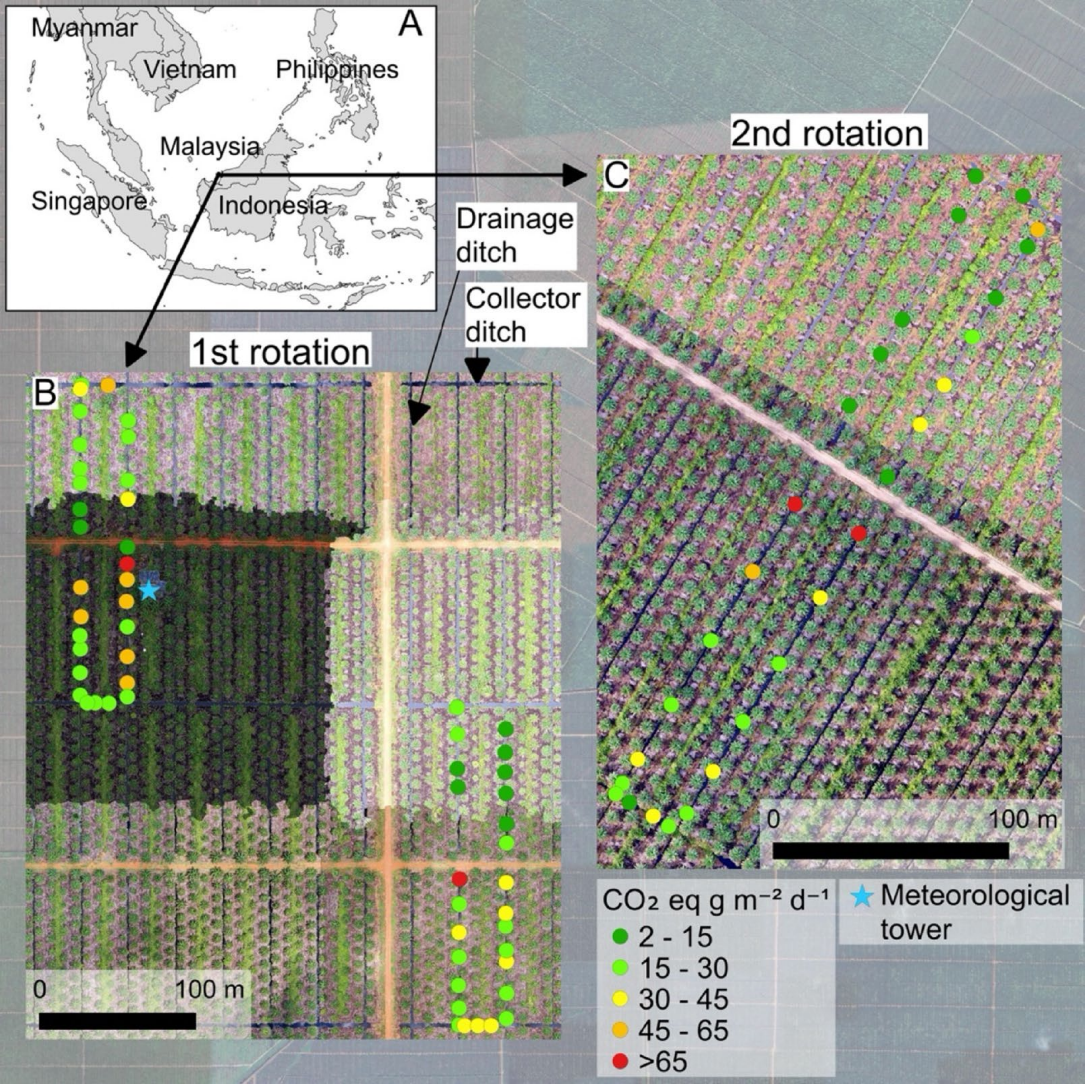
Location of the study area, in Sarawak, Malaysia (**A**) and drone images of greenhouse gas flux sampling locations in plantations marked as the first (**B**) and second (**C**) rotations. Emissions factors for each sampling spot are shown with dots, where it each dot value is expressed as g CO_2_ eq m^−2^ d^−1^ accounting for emissions of CO_2_, CH_4_, and N_2_O. Figure was created using QGIS Software (v3.10, https://qgis.org) with background map data © 2024 Google.

In addition, the current upscaling of ditch emissions is based on what fraction of the drained peatland soil area ditches occupy (“Frac_ditch_”^19^). While the 2013 Wetland Supplement^19^ provides a default fraction (0.02 for drained tropical peatlands), it also states it is “good practice” to develop country-specific ditch fractions. Therefore, current knowledge about global ditch GHG fluxes and their relative coverage of the drainage landscape are both minimal and biased towards temperate and boreal regions^23–25^. This study aims to quantify the CO_2_-equivalent GHG fluxes from drainage ditches in oil palm plantations by directly measuring GHG fluxes in a tropical peatland in Borneo, Malaysia using spatially replicated sampling at two sites (Fig. 1). Our objective with this dataset is to quantify the magnitude of CO_2_, CH_4_ and N_2_O emissions from plantation ditches and to emphasize the critical need for continuous, long-term measurements to capture the full seasonal and interannual variability of GHG fluxes in tropical regions. While geographically and temporally limited, this dataset provides a preliminary estimate and underscores the significant data gaps that exist for tropical fluxes. By leveraging high-precision analyzers and targeted chamber deployments, our sampling approach enables accurate detection of even very small GHG fluxes, including CH_4_ ebullitive events, which are often underrepresented in short-term studies. In addition, we applied innovative image processing techniques to quantify ditch water surface area, thereby improving the accuracy of regional GHG emission estimates and helping to reduce uncertainties in emission factors. Although we upscaled our results to include both first- and second-rotation plantation areas, substantial uncertainty remains regarding how emissions may evolve as these landscapes mature or are replanted. Finally, this study aims is to advance our understanding of the drivers behind spatial and temporal variability in GHG emissions. These insights are essential for improving future predictions, particularly when only small areas are sampled.

## Methods

### Study site and sampling locations

The study site is located in Betong division of Sarawak, Malaysia (Fig. 1A). The first plantation (1° 23′ 59.22″ N, 111° 24′ 6.73″ E), referred to as “first rotation”, was converted from a secondary tropical peat swamp forest to oil palm plantation in 2018 (Fig. 1B). More information on this site can be found in Kiew et al.^26^. The second plantation (1° 24′ 30.19″ N, 111° 25′ 58.86″ E), established in 2002 and referred to as “second rotation” is in its second rotation, having been replanted with new palms in 2020 after the original palms reached the end of their productive lifespan, a common practice in oil palm cultivation (Fig. 1C). Water gates regulate the groundwater levels in the plantations. We selected eight drainage ditches and four collector ditches in the first rotation, and four drainage ditches, and two collector ditches in the second rotation (Fig. 1B and C). Drainage ditches are designed to remove excess water from the land and channel it towards the collector ditches. In contrast, collector ditches serve as the primary conduits that gather water from multiple drainage ditches and direct it away from the area.

### Greenhouse gas measurements, flux calculations, and water sampling

We sampled the ditches during the daytime (approx. 8:00 a.m. to 3:00 p.m.) from April 13 to April 16, 2023. Each gas sampling spot was marked with a Global Positioning System (GPS) device. Sampling at each point consisted of a 5-min deployment of an opaque floating chamber connected to a portable GHG analyzer (LI-7810 for CO_2_/CH_4_ and LI-7820 for N_2_O, LICOR Biosciences, NE, USA). Both analyzers were factory-calibrated prior to expedition. The chamber volume was 0.065 m^3^, covering an area of 0.20 m^2^. A polystyrene plate was used to float the chamber, and two 12 V fans provided air circulation in the chamber. After each chamber deployment, we verified that all three GHGs were close to ambient air concentration at the beginning of measurement and in case of a bias, a repetitive measurement was conducted. The analyzer recirculated the air in the chamber at a rate of 0.25 L min^−1^ and recorded CO_2_, CH_4,_ and N_2_O concentrations at a frequency of 1 Hz. Following flux measurements, ditch width was measured using a Bosch GLM40 laser measuring device. Water level, pH, oxidation–reduction potential (ORP), temperature, oxygen concentration, electrical conductivity and turbidity were recorded using a portable YSI ProDSS meter (YSI Incorporated, Yellow Springs, OH, USA).

During the initial quality control, we removed gas concentration values (< 1% from the collected data) when the analyzer cavity pressure (~ 39 kPa) and cavity temperature (~ 55 ℃) were outside their operating values. The approach of Villa et al.^20^ was used to partition ebullitive and diffusive CH_4_ fluxes. Accordingly, we plotted CH_4_ concentrations versus time for each chamber deployment and visually assessed the concentration time series to identify those measurements that lacked a sudden increase, which is characteristic for bubbling events. We used data from these chambers to calculate the maximum rate of change of CH_4_ concentrations over time and assumed that any increase above this maximum was caused by ebullition. This empirical bubbling threshold in the current study was 0.16 µmol mol^−1^ s^−1^. For the CO_2_ and N_2_O flux calculations, we determined the slope of the linear regression of the change in gas concentration over the measurement period. The quality of each measurement session was validated using the R^2^ value of the linear regression. CO_2_ flux values were accepted if the R^2^ value of the slope exceeded 0.9, and N_2_O flux values were accepted if the R^2^ value exceeded 0.5. Since the N_2_O flux was very low in most sampling spots (deviated around ambient levels), we accepted the lower R^2^ for N_2_O flux calculations when the CO_2_ slope R^2^ value exceeded 0.9. Since the CO_2_ emissions are larger and more stable, it ensures that the chamber is well sealed and low N_2_O fluxes are not due to leakage of the chamber. Diffusive CH_4_ flux values were accepted if the R^2^ exceeded 0.9. A total of 86 fluxes during the three-day measurement campaign passed the quality control and were used in further analyses.

### Remote sensing imagery

To measure ditch surface water area, we used drone imagery collected for each site with a DJI Mavic 2 Pro camera drone on 23–28 October 2023 and provided by Sarawak Tropical Peat Research Institute. These data were collected in red, green, and blue (RGB) electromagnetic regions at an altitude of 60 m and ground sampling distance of 1.41 cm/pixel. Data were provided as mosaics of individual photo tiles (collected as 500 m × 200 m footprints in first rotation and 516 m × 312 m footprints in second rotation with 70% tile overlap for each site), covering ~ 1600 m × 2000 m area at first and ~ 650 m × 1100 m at second rotation. These site mosaic images had 3.30 cm and 3.58 cm spatial resolution for first rotation and second rotation, respectively. However, these mosaics were spectrally highly heterogeneous (Fig. 1) which would make it difficult to implement with a consistent water mapping approach for the full spatial extent of each image. Thus, to facilitate surface water mapping, from each image, we selected a subset of the site area with visually consistent spectral characteristics covering 61.2 ha and 16.8 ha for first rotation and second rotation, respectively.

### Mapping surface water via image classification

We delineated surface water for each image subset using object-based image classification in eCognition Developer v. 9.4 (Trimble Inc.) software. Due to spectral differences between site-specific images, a custom workflow (eCognition “rule set”) was developed for each site separately, following similar general steps.

#### Segmentation

We applied multi-resolution segmentation^27^ to generate primitive objects as mapping units. These objects were large enough to smooth local spectral noise, but small enough to capture narrow channels and small sections of open water under overhanging vegetation. A scale parameter of 40 was used with low values for shape and compactness (0.1–0.2).

#### Classification

We categorized water, vegetation and soil objects into different classes based on thresholds for different spectral features at the object level. These included red, green, and blue spectral means, and standard deviations at the object level, as well as difference- and ratio-based spectral indices that are sensitive to contrasts between these cover types (e.g., normalized differences between blue and red, between green and red signals, the ratio of blue and red difference to green, and the Excess Green Index^28,29^.

The specific steps and thresholds varied by site. For example, at the First Rotation site, where the water exhibited high spectral heterogeneity, we classified dark and light water objects separately before merging them into a single water class.

#### Region-growing process

We implemented an iterative region growing process to refine the water boundaries. Objects that were not yet classified but were adjacent to already assigned water features were segmented into finer primitive objects and evaluated for merging with the water if the spectral differences to the existing water did not exceed the allowed thresholds. This process was continued until no suitable candidates remained. Finally, all objects classified as water were merged into a single region.

#### Error assessment

The visual inspection revealed that most of the surface water areas of both the wide and narrow channels were successfully mapped. However, there were also mapping errors:

i. Inability to recognize some highly turbid areas of channels.
ii. Incorrect classification of very dark soil and tree shadow areas as water.
iii. Inability to detect channel sections obstructed by overhanging vegetation.

### Improved estimation of surface water coverage

To eliminate the mapping errors, we adopted a two-step approach:

#### Cross-sectional analysis

The mapped waterbody features were exported as Esri shapefiles in ArcGIS Desktop v.10.8.2 (Esri Inc.). We used the “Create Fishnet” tool to create gridlines equally spaced perpendicular to the water channel bodies at each site. These grid lines were intersected with the mapped water areas to obtain cross-section lines representing the width of the channels. We quantified the summary statistics of these widths separately for two types of channels at each site:

(i) Collector ditches running E-W direction at the First Rotation site (major collector ditch) and both NE-SW and NW–SE directions (major and minor collector ditches, respectively) at the Second Rotation site (Table 1);
(ii) Drainage ditches running N-S direction at the First Rotation site (wider major drainage ditches and narrower minor drainage ditches, Table 1) and NE-SW at the Second Rotation site (at the latter site, drainage ditches were split by the roads into longer and shorter sections which we measured separately, Table 1).

**Table 1:** Ditch statistics and surface water area estimates.

Ditch type serving farm subset	N	Direction	Measured length, m	Median width, m	Total ditch area, m^2^	Farm area subset, m^2^	Surface water area, m^2^/ha
First rotation							
Major collector ditch	6	E–W	500	2.00	5,755.60	168,479.03	341.62
Major drainage ditch	3	N–S	200	2.37			
Minor drainage ditch	192	N–S	97	0.83			
Second rotation							
Major collector ditch	1	NW–SE	540	1.69	23,751.14	612,000.00	388.09
Minor collector ditch	1	NE–SW	304	1.69			
Drainage ditch, longer section	17	NE–SW	158	0.86			
Drainage ditch, shorter section	17	NE–SW	137	0.86			

#### Manual measurement and area calculation

The lengths of the different channel types (longer narrow, shorter narrow, longer wide and shorter wide) were measured manually from the drone raw images using the ruler tool in ArcGIS Desktop. We counted the number of channels for each type within the subset of each site.

To estimate the area of each channel type, we multiplied its median width by the manually measured length and the number of these channels in each site’s image subset. The total area of all individual channels within each site’s subset was then summed and divided by the total area of the landscape represented by each subset to obtain a scaled measurement of m^2^ of surface water per hectare.

### Comparison with Frac_ditch_ values

We compared our results with the fraction of ditches (Frac_ditch_), a measure of the proportion of landscape area occupied by ditches and canals^30^. Frac_ditch_ values for tropical regions published by Kent^16^ and Manning et al.^17^ ranged from 0.01 to 0.09. Our values (341.62 m^2^ ha^−1^ = 0.034 and 388.09 m^2^ ha^−1^ = 0.039; Table 1) fall within this range.

### Data analysis and calculation of emission factors

The Shapiro–Wilk test was used to check the normality of gas fluxes. As the distribution of the data was skewed, we used the non-parametric Mann–Whitney *U* test to analyze the CO_2_, CH_4_, and N_2_O flux differences between the two rotations. Spearman’s rank order correlation analyses were conducted to observe the relationships between gas fluxes and water chemical and physical parameters. Since the data were skewed, we used the median values of measurements for upscaling to estimate daily fluxes and calculated CO_2_ equivalents using equivalent values for CH_4_ and N_2_O of 34 and 298, respectively^31^. For CH_4_ upscaling, we used total flux (diffusive + ebullition) because ebullition dominated over diffusive flux and occurred in most of the sampling spots. Using only diffusive flux would potentially underestimate the total CH_4_ emission by at least half. Then, we multiplied the median daily flux values with ditch surface area per ha estimated by the surface water mapping (Table 1), which was 341.62 m^2^ in the first rotation and 388.09 m^2^ in the second rotation per ha of plantation area. Furthermore, we estimated daily GHG fluxes from ditches across the whole Sarawak region, which includes a total of 460,000 ha of oil plantations^32^. Although our measurement was limited to a few days, we leveraged the relatively stable tropical climate, where variations across seasons tend to be less extreme than in temperate regions. All statistical analyses were conducted, and figures were created in R using the following packages: *ggplot2*^33^, *dplyr*^34^, and *corrplot*^35^. Figure 1 was created using QGIS v3.10^36^.

## Results

### Local survey of ditch GHG fluxes

**Fig. 2:**
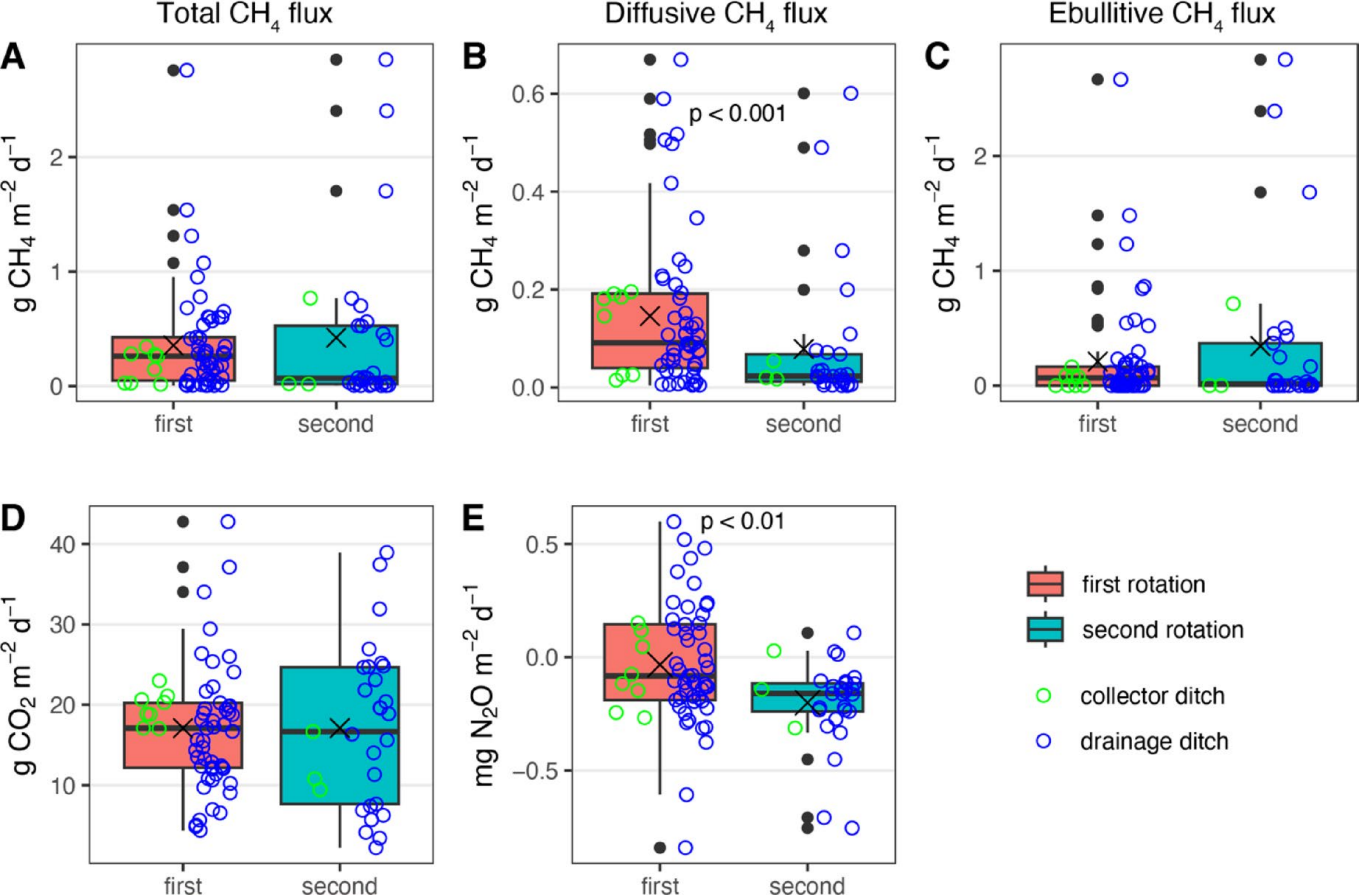
Box plots of total CH_4_ fluxes (**A**), diffusive CH_4_ fluxes (**B**), ebullitive CH_4_ fluxes (**C**), CO_2_ fluxes (**D**), and N_2_O fluxes (**E**) from first (n = 57) and second (n = 29) rotation for collector ditches and drainage ditches in Sarawak, Malaysia. Boxes represent medians and interquartile ranges, whiskers mark minimum and maximum values. Also shown are mean fluxes (x) and outliers. Green circles represent fluxes from collector ditches and blue circles from drainage ditches. Note that CH_4_ y-axis scales differ between panels A, B, and C, where diffusive CH_4_ fluxes are shown on smaller scale.

The median GHG fluxes, combined over drainage and collector ditches across two rotations were 0.18 (range: 0.004 to 2.83) g CH_4_ m^−2^ d^−1^, 17.1 (range: 2.2 to 42.8) g CO_2_ m^−2^ d^−1^, and − 0.12 (range: − 0.53 to 0.38) mg N_2_O m^−2^ d^−1^. The median ebullitive CH_4_ flux was 0.07 (range: 0 to 2.67) g CH_4_ m^−2^ d^−1^ from the first rotation, and 0.01 (range: 0 to 2.84) g CH_4_ m^−2^ d^−1^ from the second rotation (Table 2). Among the 86 sampling points, ebullition events were observed at 50 points, indicating clear dominance over diffusive CH_4_ flux (Fig. 2c). The total CH_4_ flux, ebullitive CH_4_ flux, and median CO_2_ flux did not exhibit significant variations between rotations or across different ditch types (Fig. 2a, c, d). However, the diffusive CH_4_ flux was significantly higher in the first rotation (Fig. 2b; W = 1189, p < 0.001). Additionally, the N_2_O flux was significantly lower in the second rotation (Fig. 2e; W = 1144, p = 0.01).

**Table 2:** The median/mean, and range of greenhouse gas fluxes of carbon dioxide (CO_2_), methane (CH_4_), and nitrous oxide (N_2_O), and water chemical-physical properties from drainage ditches in first and second rotation in Sarawak, Malaysia.

	First rotation (n = 57)		Second rotation (n = 29)		Combined (n = 86)		
	Median/mean	Range	Median/mean	Range	Median/mean		Range
CO_2_ g m^−2^ d^−1^	17.1/17.1	4.4–42.8	16.7/17.1	2.2–38.9	17.1/17.1		2.2–42.8
CH_4_ total g m^−2^ d^−1^	0.19/0.34	0.004–2.66	0.07/0.42	0.004–2.83	0.18/0.37		0.004–2.83
CH_4_ diffusive g m^−2^ d^−1^	0.09/0.14	0.004–0.67	0.02/0.08	0.004–0.6	0.06/0.12		0.004–0.67
CH_4_ ebullitive g m^−2^ d^−1^	0.07/0.20	0–2.67	0.01/0.34	0–2.82	0.04/0.25		0–2.82
N_2_O mg m^−2^ d^−1^	− 0.08/− 0.03	− 0.53–0.38	− 0.16/− 0.20	− 0.16–0.07	− 0.12/− 0.09		− 0.53–0.38
pH	3.60/3.62	3.54–3.92	3.70/3.73	3.61–4.07	3.63/3.66		3.54–4.07
Oxidation–reduction potential	346.0/338.5	175.6–452.2	306.0/284.8	156–366.1	323.4/320.4		156–425.2
Water temperature (℃)	29.9/30.1	28.1–33.1	28.7/28.7	27.5–29.8	29.2/29.6		27.5–33.1
Dissolved oxygen concentration (mg L^−1^)	0.90/1.66	0.17–6.1	0.46/0.85	0.21–4.05	0.83/1.39		0.17–6.1
Electrical conductivity (µS cm^−1^)	88.0/85.7	60.8–92.5	83.8/83.8	72.3–90.9	87.3/85.1		60.8–92.5
Water depth (cm)	55.0/54.4	30–78	47.0/46.6	26–67	50.0/51.8		26–78

**Fig. 3:**
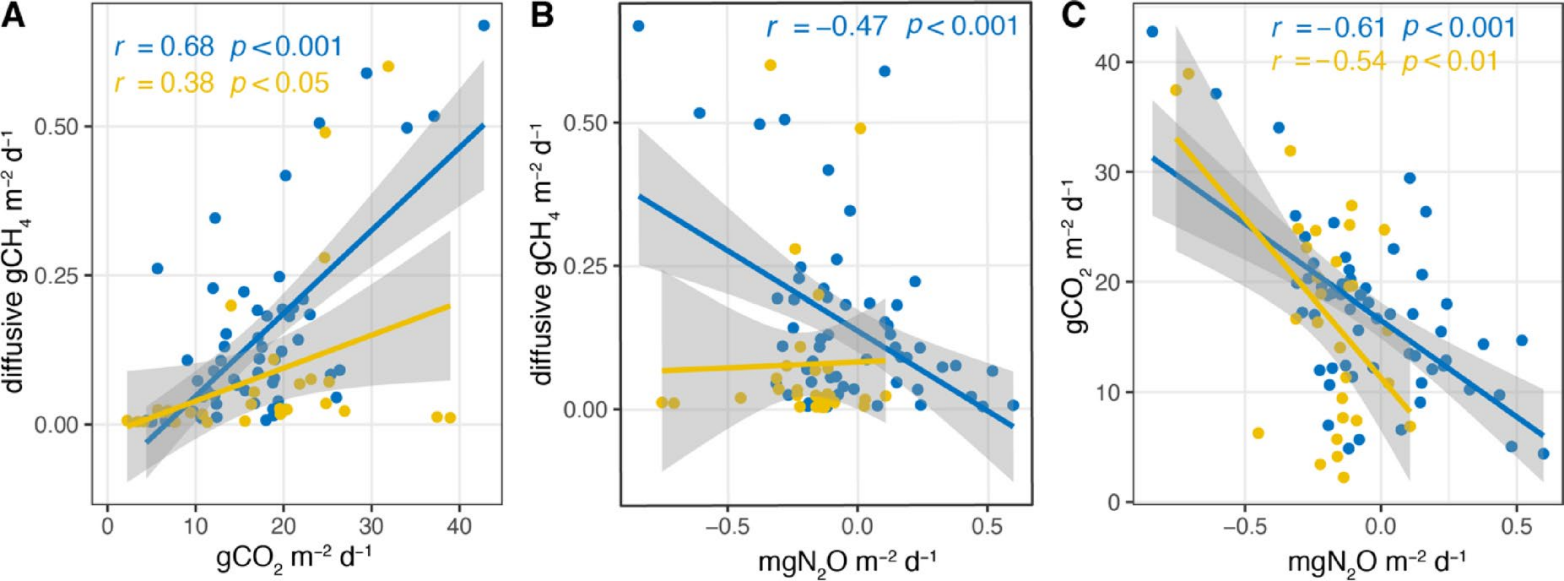
Scatterplots showing the relationships between diffusive CH_4,_ and CO_2_ flux (**A**), diffusive CH_4_ and N_2_O flux (**B**), CO_2_ and N_2_O flux (**C**) during the first and second rotations. The color of regression lines and coefficients (r) corresponds to the rotation: blue for the first rotation and yellow for the second rotations. Only statistically significant regression coefficients are displayed, with significance determined at p < 0.05. Shaded areas are 95% confidence intervals.

In both rotations, there was a significant positive correlation between diffusive CO_2_ and CH_4_ fluxes, with Pearson correlation coefficients of r = 0.68 (p < 0.001) in the first rotation and r = 0.38 (p < 0.05) in the second rotation (Fig. 3a). Additionally, a significant negative correlation was observed between CO_2_ and N_2_O fluxes, with r = − 0.61 (p < 0.001) in the first rotation and r = − 0.54 (p < 0.01) in the second rotation (Fig. 3c). Notably, a negative correlation between diffusive CH_4_ and N_2_O fluxes was only significant in the first rotation, with r = − 0.47 (p < 0.001; Fig. 3b).

**Fig. 4:**
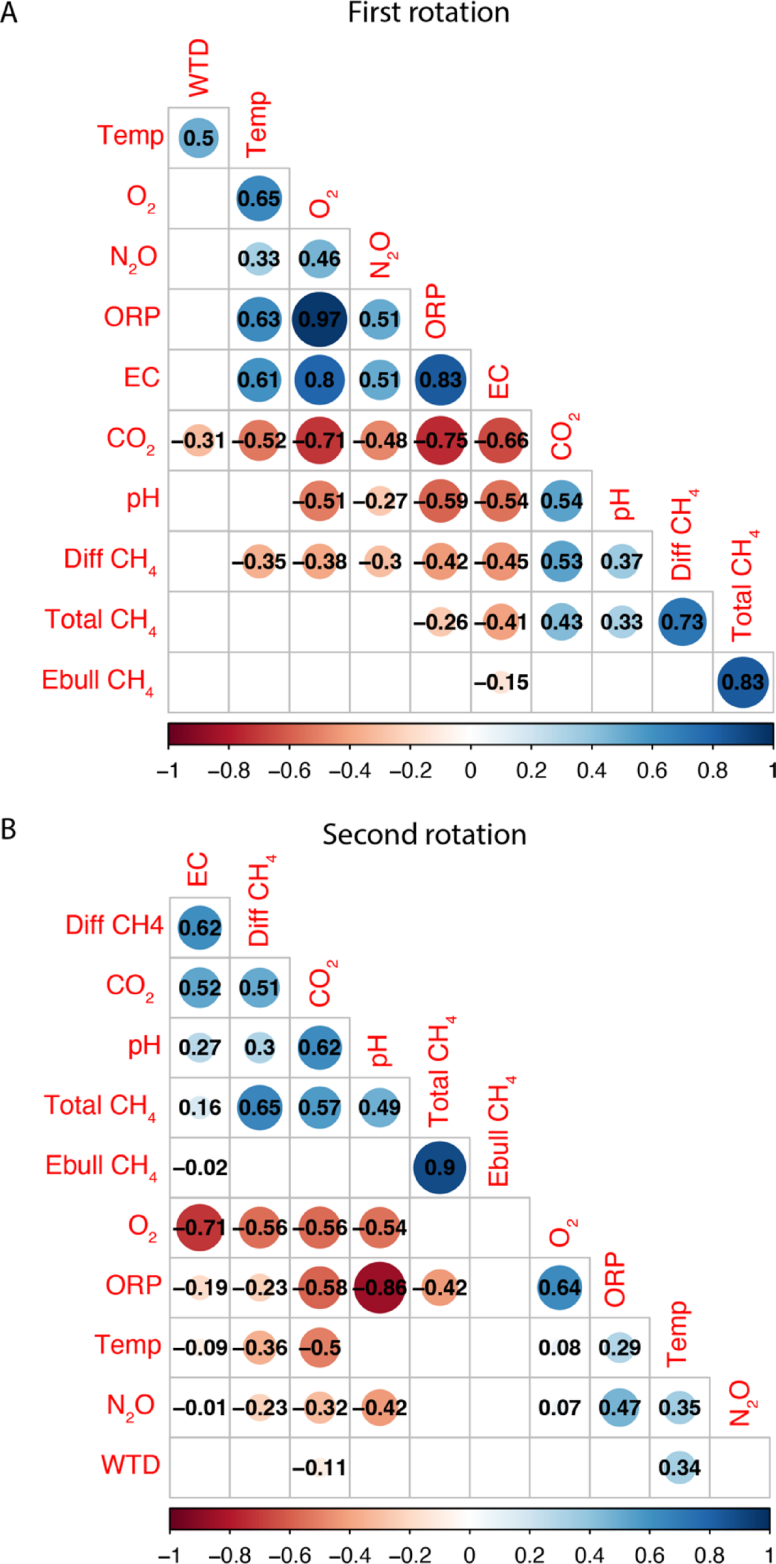
Spearman correlation matrix for greenhouse gas fluxes and water parameters in the first rotation (**A**) and second rotation (**B**) for drainage ditches in Sarawak, Malaysia. ORP = oxidation–reduction potential, WTD = water level, Diff = diffusive, Ebull = ebullitive, Temp = water temperature. Only statistically significant (p < 0.05) correlations are shown.

We observed several significant relationships between GHG fluxes and water parameters. There was a strong positive correlation between CO_2_ diffusive flux and water pH in both rotations, with Spearman correlation coefficients (Fig. 4A and B) of ρ = 0.54 for the first rotation and ρ = 0.62 for the second rotation. Conversely, CO_2_ flux in both rotations showed significant negative correlations with oxidation–reduction potential (ρ = − 0.75 and ρ = − 0.58), dissolved oxygen concentration (ρ = − 0.71 ρ = − 0.56), and water temperature (ρ = − 0.52 and ρ = − 0.5). For CH_4_ diffusive flux, significant negative correlations were found with oxidation–reduction potential (ρ = − 0.42 and ρ = − 0.23), conductivity (ρ = − 0.45 and ρ = 0.62), dissolved oxygen concentration (ρ = − 0.38 and ρ = − 0.56), and pH (ρ = − 0.37 and ρ = 0.3). In the first rotation, the N_2_O flux was positively correlated with dissolved oxygen concentration (ρ = 0.46), water temperature (ρ = 0.65), conductivity (ρ = 0.51), ORP (ρ = 0.51), and negatively correlated with water pH (ρ = − 0.27). In the second rotation, N_2_O had a negative correlation with pH (ρ = − 0.42) and a positive correlation with ORP (ρ = 0.47), and water temperature (ρ = 0.35). Scatterplots and data distribution of different variables in the first and second rotation are shown in Supplementary Fig. S1 (First rotation) and Fig. S2 (Second rotation).

### Upscaling of ditch GHG fluxes and emission factors

To upscale fluxes to one ha of drained land, we used ditch area estimations based on image classification, which were 341.62 m^2^ ha^−1^ in the first rotation and 388.09 m^2^ ha^−1^ in the second rotation. Therefore, the median CH_4_ emissions from one hectare of drained land in Sarawak plantations were 0.07 kg CH_4_ ha^−1^ d^−1^ (3.02 kg CO_2_ eq ha^−1^ d^−1^) and 5.84 kg CO_2_ ha^−1^ d^−1^ in the first rotation, and 0.02 kg CH_4_ ha^−1^ d^−1^ (1.05 kg CO_2_ eq ha^−1^ d^−1^) and 5.69 kg CO_2_ ha^−1^ d^−1^ in the second rotation. All ditches were low N_2_O sinks with the median flux of − 0.0008 kg N_2_O as CO_2_ eq ha^−1^ d^−1^ in the first rotation and − 0.001 kg N_2_O as CO_2_ eq ha^−1^ d^−1^ in the second rotation (Table 3). Collectively, the ditches in the Sarawak region (~ 460 000 ha of oil plantations) emitted 30 tons of CH₄ d^−1^ and 2868 tons of CO_2_ d^−1^. Additionally, the ditches acted as small N_2_O sinks, with a daily uptake of − 0.004 tons N_2_O d^−1^.

**Table 3:** The median ditch emission factors for both plantation rotations are expressed as kilogram CO_2_ eq ha^−1^ d^−1^, accounting for emissions of CO_2_, CH_4_, and N_2_O. The emission factors per ha of land were calculated based on ditch coverage in one ha of land, which was 341.62 m^2^ in the first rotation and 388.09 m^2^ in the second rotation.

	CO_2_ kg ha^−1^ d^−1^	CH_4_ kgCO_2_ eq ha^−1^ d^−1^	N_2_O kgCO_2_ eq ha^−1^ d^−1^	CO_2_-equivalent kgCO_2_ eq ha^−1^ d^−1^
First rotation	5.84	3.02	− 0.0008	9.51
Second rotation	5.69	1.05	− 0.001	8.99
Combined	5.84	2.78	− 0.001	9.13

## Discussion

### Local survey of ditch GHG fluxes

All drainage ditches in the oil palm plantation in Sarawak emitted substantial amounts of CO_2_ and CH_4_, while most acted as small sinks for N_2_O. The observed total CH_4_ emissions were comparable to those previously reported (0.001–1.64 g CH_4_ m^−2^ d^−1^) from drainage ditches in organic soils in Brunei^37^, Indonesia^5,16,18^, and Sarawak, Malaysia^17^ (Table 4).

**Table 4:** Mean methane (CH_4_) and nitrous oxide (N_2_O) fluxes from peatland drainage channels in South-East Asia.

Country	Setting	CH_4_flux (g CH_4_m^−2^d^−1^)	N_2_O flux (mg N_2_O m^−2^d^−1^)	References
Indonesia	Cleared, drained, and abandoned peatland	0.164	0	5
Indonesia	Acacia wood plantation, undisturbed 5 years	0.107	2.78	5
Indonesia	Acacia wood plantation, recently disturbed	0.09	0.19	5
Indonesia	Channel through degraded peatland	0.001–0.04	–	17
Indonesia	Drainage canals	0.09	–	19
Brunei	Partially drained and partially deforested peatland	0.041–0.45	–	34
Malaysia	Drainage ditch in oil palm plantation	0.136	–	18
Malaysia	Drainage ditch in oil palm plantation, 1st rotation)	0.34	− 0.03	This study
Malaysia	Drainage ditch in oil palm plantation, 2nd rotation)	0.42	− 0.20	This study

CH_4_ emissions in these ditches can occur through diffusion, ebullition, or plant-mediated transport^38^. However, in the drainage ditches of the Sarawak oil palm plantation, plant-mediated CH_4_ transport in the ditches was not a factor due to the absence of macrophyte vegetation. We however note that Manning et al.^17^ indicated CH_4_ emissions from stems of oil palms as also a significant pathway of CH_4_ emissions. The lack of vegetation in ditches effectively rules out plant-induced CH_4_ transport from ditches, leaving diffusion and ebullition as the primary pathways for CH_4_ emissions. This exclusion emphasizes the significance of chemical and physical processes in water and sediment, such as dissolved organic carbon (DOC) driven CH_4_ production identified by Manning et al.^17^, which varies based on drain type and environmental conditions. Manning et al.^17^ also found that smaller field drains in Sarawak emitted more CO_2_ than larger collection drains, as DOC first reached these field drains from the soil. This finding could suggest that ditch size and configuration affect CH_4_ flux rates in Sarawak ditches, particularly under varying hydrological conditions.

We showed that the diffusive CH_4_ flux was significantly higher in the first rotation. This can indicate a decrease in diffusive flux as the plantation ages. The lower diffusive flux in older ditches can feasibly be attributed to a lower lateral inflow of CH_4_^39^ and/or a reduction of labile organic matter in ditch sediments, which could be depleted as ditches age (e.g. as for other constructed waterbodies)^40^. This cumulative effect of organic matter decomposition and CH_4_ production over time can result in higher CH_4_ concentration in the ditch sediments and more frequent ebullitive events. Occurrences of ebullition have previously been considered episodic events that do not contribute significantly to total flux^38^. Some recent studies have even shown that ebullitive flux contribution is relatively low compared with diffusive flux from ditches^41^. However, our high-frequency sampling revealed that ebullitive flux dominated over diffusive flux in tropical ditches in organic soils. Similar results have been shown by Kiew et al.^26^, Villa et al.^20^, and Bastviken et al.^42^, who confirmed that ebullition dominated from the water surface in a temperate freshwater marsh and in lakes, respectively. It is important to note, however, that our method may under-estimate larger, more stochastic CH_4_ ebullition events, as these could be missed due to their episodic nature. Employing a bubble trap method, as suggested by Männistö et al.^43^, could improve accuracy in capturing both frequent “micro” ebullition events and the larger, less frequent ebullition events. Using bubble traps in future studies may thus help quantify the full extent of ebullitive CH_4_ emissions.

Water pH was acidic across all sampling locations and in both plantation rotations. Despite these conditions, diffusive CH_4_ fluxes showed a positive correlation with water pH. Similarly, soil pH values indicated acidic conditions, which are generally suboptimal for methanogens but may still support acid-tolerant species^44^. However, even slightly higher pH conditions can provide better conditions for methanogens that favor more neutral conditions^45^. We did not observe a relationship between CH_4_ flux and water depth, contrary to previous findings^15,46^. Water depth exhibited minimal variation, potentially obscuring clear correlations with the highly variable CH_4_ emissions, as was found in multi-site studies conducted by Knox et al.^47^. The strong negative correlation with dissolved oxygen concentration was observable in both rotations, indicating that higher oxygen levels reduce CH_4_ fluxes^48^. Therefore, we suggest that relatively high water levels, combined with elevated temperatures and low dissolved oxygen concentrations, create conditions that, in the presence of sufficient organic matter or DOC, promote anoxia and support the establishment of microbial communities favourable for CH_4_ production^49^. This was also shown by Manning et al.^17^, who observed that CH_4_ emissions responded to seasonal temperature variations, with warmer air temperatures promoting CH_4_ diffusion. Our results also indicate that when the pH is not a limiting factor for methanogenesis, the emission could be much higher in the more neutral environments that exist in organic soils^50^.

The observed negative correlation between CO_2_ flux and both water temperature and oxygen concentration suggests that CO_2_ may, in part, be a byproduct of CH_4_ production by methanogens, which is then partially oxidized as it moves through the water column^38^. During acetoclastic methanogenesis, acetate is split into CH_4_ and CO_2_, while hydrogenotrophic methanogenesis consumes CO_2_ to produce CH_4_. Thus, depending on the dominant pathway, methanogenesis can result in net CO_2_ production. CH_4_ oxidation by methanotrophs to CO_2_ can also be particularly significant in acidic conditions, where methanotrophs are often more tolerant than methanogens^51^. The positive correlation between CO_2_ and CH_4_ flux implies that both CH_4_ and CO_2_ fluxes may be interconnected, as both gases emerge from the same decomposing organic material under anoxic conditions^52,53^. Therefore, both methanogenesis and ecosystem respiration can be influenced by the same environmental factors^54^ and fueled by common carbon substrates, leading to supersaturation of dissolved CO_2_ and CH_4_^55^. A similar positive relationship between CH_4_ and CO_2_ flux in forest drainage ditches was observed by Peacock et al.^15^. Perryman et al.^18^ clearly showed that a large part of CH_4_ in tropical peatland drainage canals might be oxidized. Additionally, as water temperatures increase, CO_2_ solubility decreases, which results in less CO_2_ remaining dissolved and thereby leads to higher emissions^56^.

Regarding N_2_O, we predominantly observed negative fluxes, indicating net consumption, likely through denitrification processes in sediments or water columns, which is consistent with previous findings^57,58^. N_2_O flux showed a strong negative correlation with water pH but a positive correlation with oxygen concentration, temperature, and ORP. Like methanogenesis, denitrification processes can be inhibited at low pH^59^, leading to minimal N_2_O production. Under highly acidic conditions, dissimilatory nitrate reduction to ammonium (DNRA) may dominate, converting nitrate directly to ammonium rather than producing N_2_O or N_2_^60^. Although low pH may limit the entire denitrification process, some N_2_O reduction was observable at several gas flux sampling locations, suggesting that small-scale consumption can still occur. For instance, complete denitrification in the water column can happen at very low oxygen concentrations^61^ and at optimal temperatures for denitrification, typically between 25 and 35 °C^59,62^.

The negative correlation between diffusive CH_4_ and N_2_O flux in the first rotation suggests that, although environmental conditions might favor both methanogenesis and denitrification, methanogenesis may dominate. When conditions favor methanogenesis, nitrate and other oxidized nitrogen compounds are often depleted, reducing the potential for denitrification, and thus limiting N_2_O production^63,64^. The observed negative correlation between CO_2_ and N_2_O fluxes likely reflects shifts in microbial processes under varying redox conditions in the ditches. Under more anoxic conditions denitrification may increase and potentially lead to greater N_2_O production, however, if conditions become highly reduced complete denitrification may also occur^65^.

### Upscaling the fluxes

Our findings also provide important insights into the strengths and challenges of using remote sensing in monitoring and upscaling fluxes in oil palm plantations. Drone images are very promising in this regard because their high spatial resolution allows delineating complex landscape objects and boundaries (e.g., between water and palm tree canopies), while the timing of flights can be customized according to project schedules and constraints. In our landscape study such data made it possible to detect variation in the width of narrow ditches and incorporate that information into surface water estimation, which would not be feasible at ≥ 3 m of most non-commercially available satellite products.

At the same time, several important limitations should be acknowledged and considered in the future efforts. First, basic true-color, or RGB imagery (composed of signals in red, green, and blue electromagnetic regions) is highly limited in the capacity to differentiate between water and non-water features, in contrast to successful detection of plantation trees^66,67^. Here this challenge was further amplified by water turbidity (increasing its similarity with soil) and by spectral non-uniformity of individual image scenes collected and stitched by the data provider. This issue could be remedied by using drone cameras with near-infrared or thermal sensing capacity due to high contrast in near-infrared reflectance between land and water; however, cost of multi-spectral instruments beyond common RGB cameras may be an issue when monitoring budgets are limited^68,69^. Further, even with near-infrared and thermal data, spectral artifacts such as dark and cool shadows can still pose challenges due to confusion with water and may require corrections based on object shape, spatial adjacency to trees, etc.

Second, estimation of surface water areas may be sensitive to landscape configuration of the site. Floating or overhanging vegetation (e.g., large palm canopies) can make portions of water channels not identifiable as water, despite the contributions of those portions to CH_4_ fluxes. We navigated this issue by computing channel area based on measured lengths and statistical distributions of widths, rather than net mapped water area alone; however, such an approach has its own uncertainty associated with statistical sampling.

Based on these insights, future work can facilitate the upscaling of plantation ditch fluxes by (1) procuring imagery with high sensitivity to water-land and water-vegetation contrasts, such as a combination of visible and near-infrared and/or thermal sensors; (2) aiming for high quality data acquisition with robust radiometric calibration between drone image tiles to achieve uniform spectral properties of similar cover types across the whole surveyed area, and (3) obtaining accurate ground-level information on ditch design, such as length to help refine mapped surface water estimates and correct for overhanging vegetation.

### Regional ditch greenhouse gas emissions estimates

The land use conversion of peat swamp forests to oil palm plantations significantly increases GHG fluxes. Over the past decades, the extent of oil palm plantations has been increasing substantially in the entire Southeast Asian region, and the largest development took place between 2010 and 2017^6^. Data from Peninsular and East Malaysia, Kalimantan, Sumatra, and Brunei showed that the annual GHG balance from oil palm plantations was 258.5 tCO_2_ eq ha^−1^ yr^−1^ in young oil palm plantations and 97.4 tCO_2_ eq ha^−1^ yr^−1^ in mature oil palm plantations^13^. These high numbers are mostly driven by very large CO_2_ and N_2_O emissions. The total GHG balance from degraded and *Acacia* plantations in Indonesia was 45.1 and 35.2 tCO_2_ eq ha^−1^ yr^−1^^70^. These estimations, on the other hand, are based on measurements from drained soil and do not consider the emissions from drainage ditches. Our results showed that the daily emission factor for drainage ditches in plantations was 9.13 kgCO_2_ eq ha^−1^ d^−1^, which originates from a relatively minor fraction of the landscape. Free surface water in the ditches constitutes only approximately 3.6% per ha of land, nearly double the default values provided by the IPCC 2013 Wetland Supplement for the tropical climate zone^19^. It has been shown that peatland ditches in northern regions are landscape-scale CH_4_ emission hot spots, but not for CO_2_^71^. In our study, we showed that this is not the case for ditches in tropical regions, where ditches on organic soils are both large emitters of CH_4_ and CO_2_, as was also shown by Manning et al.^17^. Our calculations based on median fluxes showed that ditch emissions contribute about 4% of the annual GHG emissions in first rotation and about 10% in the second rotation per ha of land. The percentages were calculated based on GHG balance estimations from Cooper et al.^13^ for young and mature planatations. Since the fluxes from our study fell into the same range as other previously published studies (Table 4) we assume that these proportions could be similar in other regions as well. On the other hand, since our collected data was skewed, we used median values for calculations. However, mean fluxes were almost two times higher than median values, indicating that we might underestimate the actual emissions and therefore long-term continuous measurements over multiple areas are needed for more precise flux estimations.

In addition, our short-term campaign suggests that, at the regional scale, ditch fluxes can represent important emission pathways that substantially contribute to the total land-based GHG budget. We also saw that the age of the plantation does not reduce the total GHG flux from ditches, although the emission from dry land may decrease substantially over time^13^. Therefore, we can assume that even though many of the plantations are still under first rotation, the replanting of oil palms or the ages of the ditches do not influence GHG fluxes if organic soils remain and are not completely oxidized. Finally, we acknowledge several limitations in our study. First, our sampling was conducted over only three days, capturing a transitional period from the drier season to the wetter season. As Manning et al.^17^ demonstrated, CH_4_ emissions can follow a seasonal trend. While seasonal effects are less pronounced in tropical regions compared to boreal or temperate zones, they can still influence GHG production and consumption processes. Additionally, our dataset was skewed, leading us to use median flux values for upscaling. While this approach mitigates the influence of extreme values, it is worth noting that the mean flux values were nearly twice as high as the median. As a result, our flux estimates likely underestimate rather than overestimate the total emissions. These limitations underscore the need for long-term, continuous measurements of GHG fluxes from tropical drainage ditches. Automated floating chambers capable of continuous data collection could provide critical insights into the temporal dynamics of GHG fluxes, addressing significant knowledge gaps in this area.

## Conclusions

This study demonstrates that drainage ditches in tropical oil palm plantations are significant and under-studied sources of CO_2_ and CH_4_ emissions. We saw that these emissions do not decline as plantations age but persist over time, with ebullitive CH_4_ fluxes dominating and increasing slightly with plantation maturity. The minimal N_2_O fluxes observed likely result from the acidic conditions, which suppress denitrification, although plantations on peat soils with higher pH levels could see elevated N_2_O emissions, highlighting pH as a key factor. The study also underscores the high variability of GHG fluxes driven by complex environmental interactions at both local and regional levels. These findings emphasize the need for future research and models to incorporate the dynamic spatio-temporal factors influencing GHG emissions to better assess and manage the environmental impact of tropical oil palm plantations.

## Supplementary Information

Below is the link to the electronic supplementary material.

Supplementary Material 1

Supplementary Material 2

## Data Availability

Data is provided within the supplementary information files.
