# Supplementary material for: Greenhouse gas emissions from ditches in oil palm plantations on tropical peatlands in Malaysia
Source: Sci Rep. 2025 Oct 23;15:37126. doi: 10.1038/s41598-025-21094-3 (PMC12550050; doi:10.1038/s41598-025-21094-3)
Supplement: Supplementary file 2 — Supplementary Material 2 [file 41598_2025_21094_MOESM2_ESM.docx]

**SUPPLEMENTARY**

**GREENHOUSE GAS EMISSIONS FROM DITCHES IN OIL PALM PLANTATIONS ON TROPICAL PEATLANDS**

Kuno Kasak,^1,2*^ Iryna Dronova,^2^ Kaido Soosaar,^1^ Lulie Melling,^3^ Wong Guan Xhuan,^3^ Faustina Sangok,^3^ Reti Ranniku,^1^ Jorge A. Villa,^4^ Sheel Bansal,^5^ Michael Peacock,^6,7^ Ülo Mander ^1^

^1^ University of Tartu, Department of Geography, Tartu, Estonia

^2^ Department of Environmental Science, Policy, and Management, University of California, Berkeley, USA

^3^ Sarawak Tropical Peat Research Institute, Sarawak, Malaysia

^4^ University of Louisiana at Lafayette, LA, USA

^5^ U.S. Geological Survey, Northern Prairie Wildlife Research Center, Jamestown, ND, USA

^6^ Department of Aquatic Sciences and Assessment, Swedish University of Agricultural Sciences, Uppsala, Sweden

^7^ Department of Geography and Planning, University of Liverpool, UK

*email: [kuno.kasak@ut.ee](mailto:kuno.kasak@ut.ee)


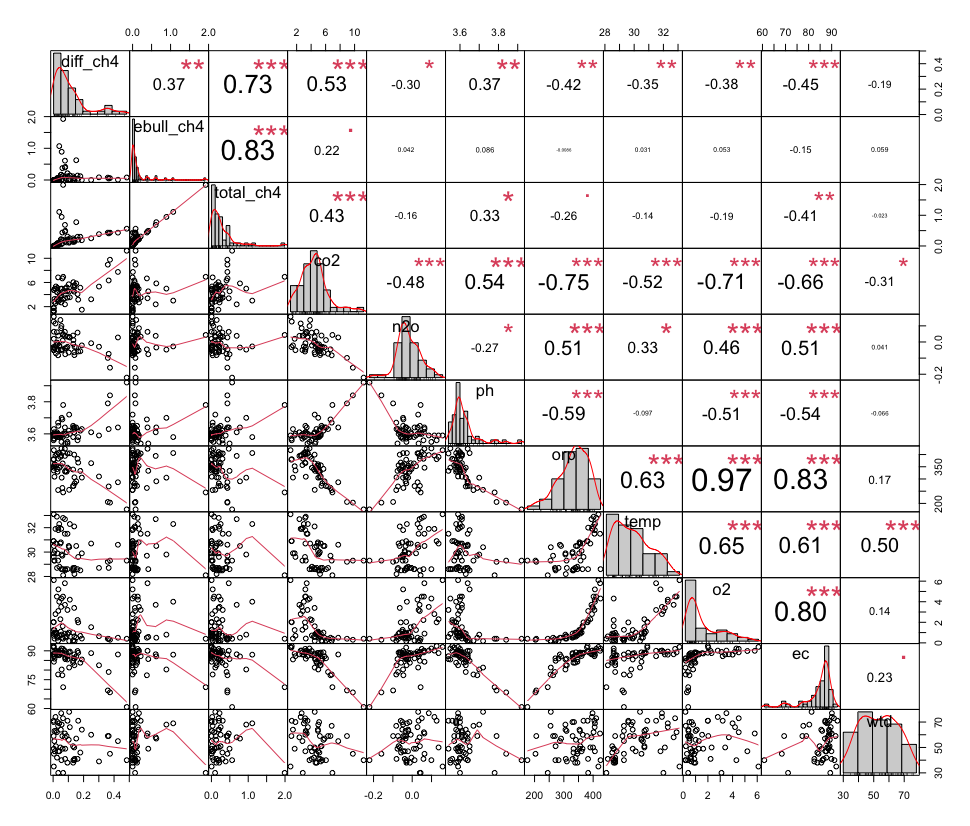


Figure S1. Spearman correlation matrix in the first rotation with scatterplots and histograms


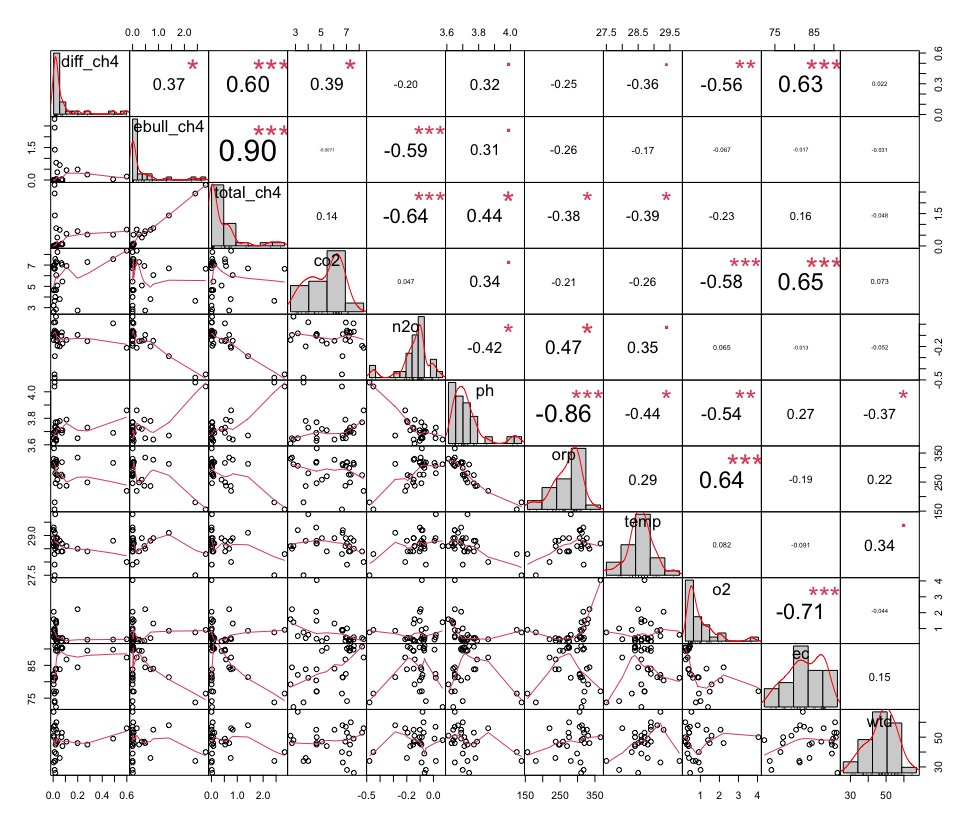


Figure S2. Spearman correlation matrix in the second rotation with scatterplots and histograms
